# Supplementary figures and images for: Effective Natural Killer Cell Degranulation Is an Essential Key in COVID-19 Evolution
Source: Int J Mol Sci. 2022 Jun 13;23(12):6577. doi: 10.3390/ijms23126577 (PMC9224310; doi:10.3390/ijms23126577)

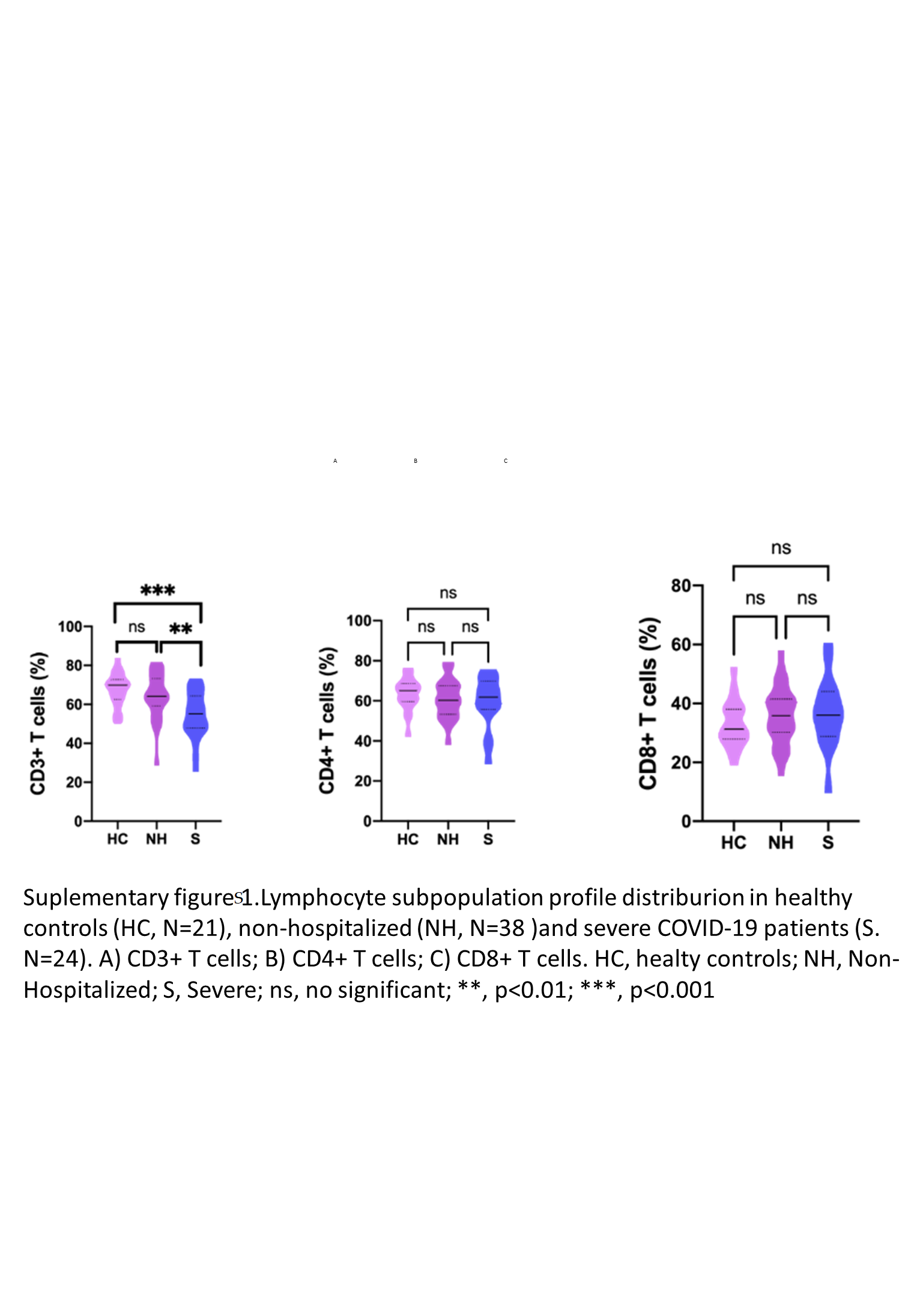

Supplement: Supplementary file 1 [file ijms-23-06577-s001.zip › Diapositiva1.TIF]

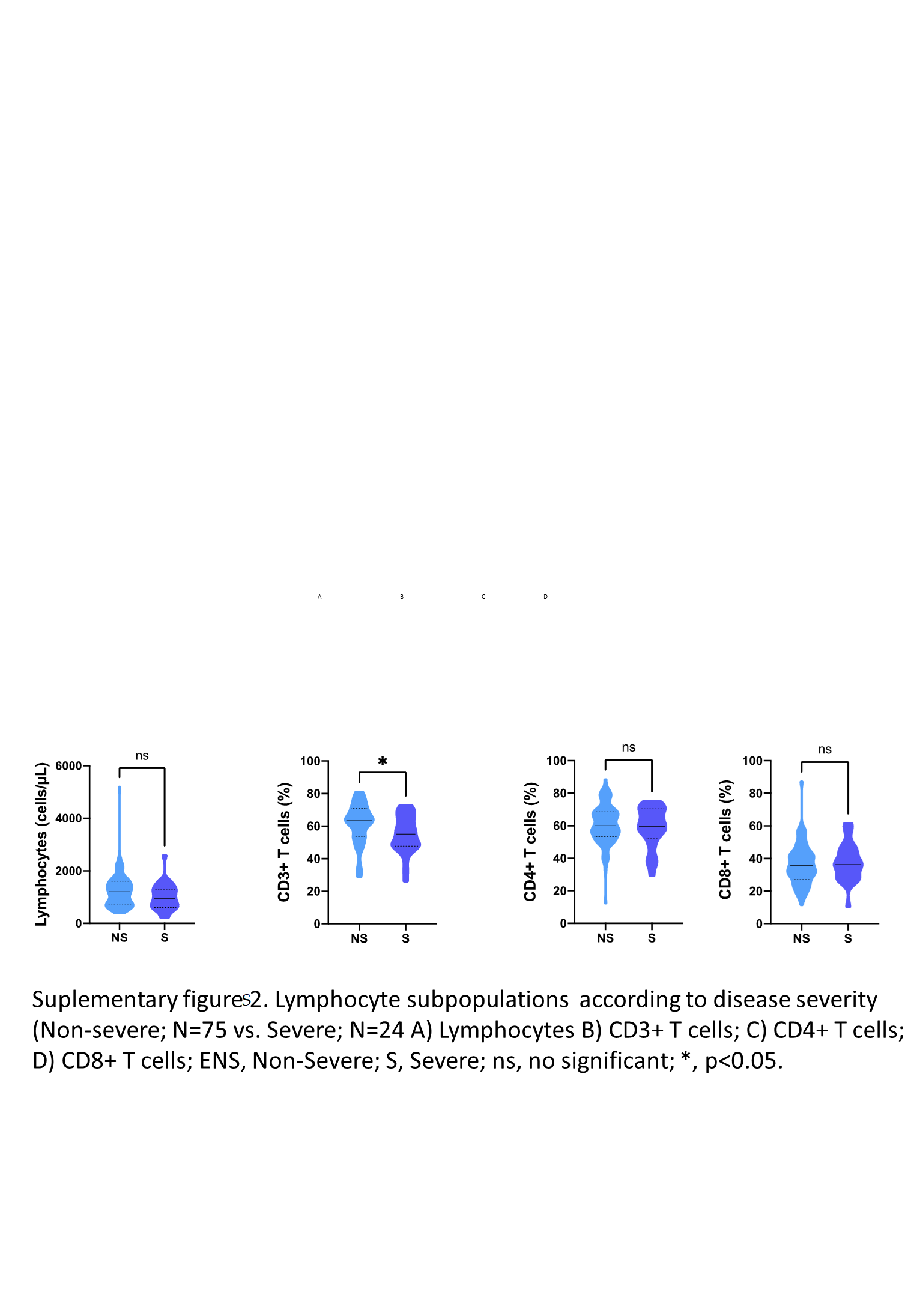

Supplement: Supplementary file 1 [file ijms-23-06577-s001.zip › Diapositiva2.TIF]

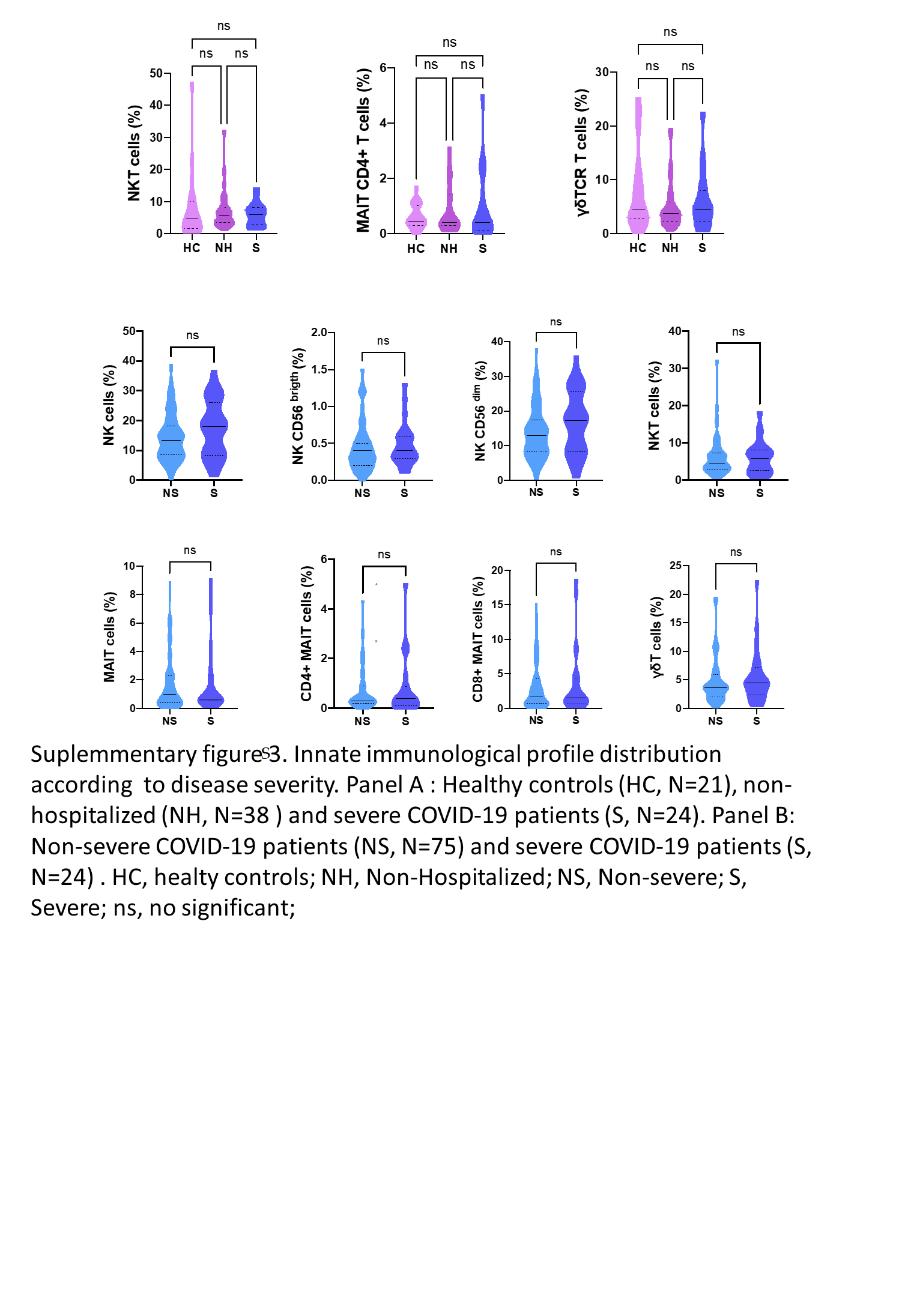

Supplement: Supplementary file 1 [file ijms-23-06577-s001.zip › Diapositiva3.TIF]

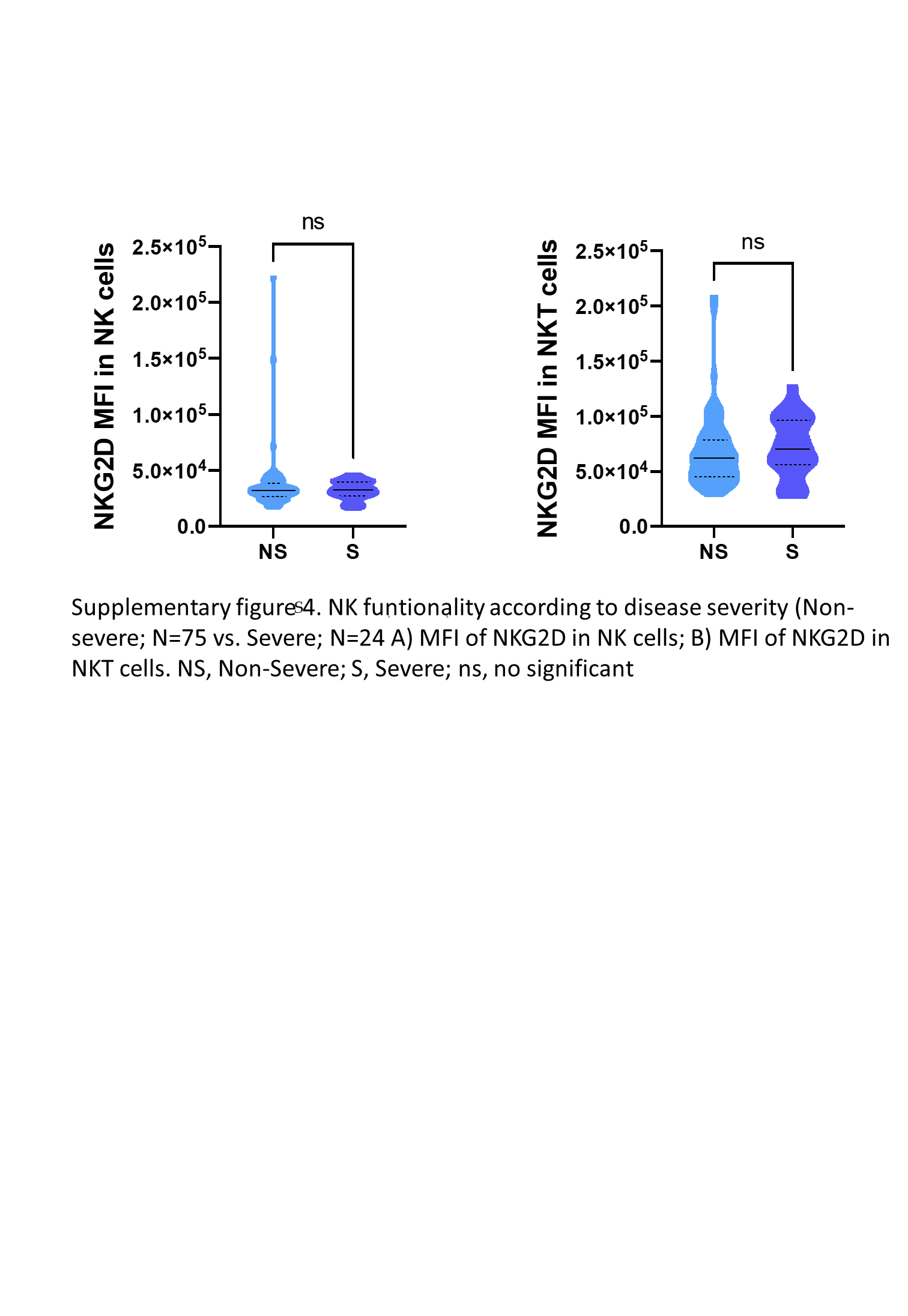

Supplement: Supplementary file 1 [file ijms-23-06577-s001.zip › Diapositiva4.TIF]

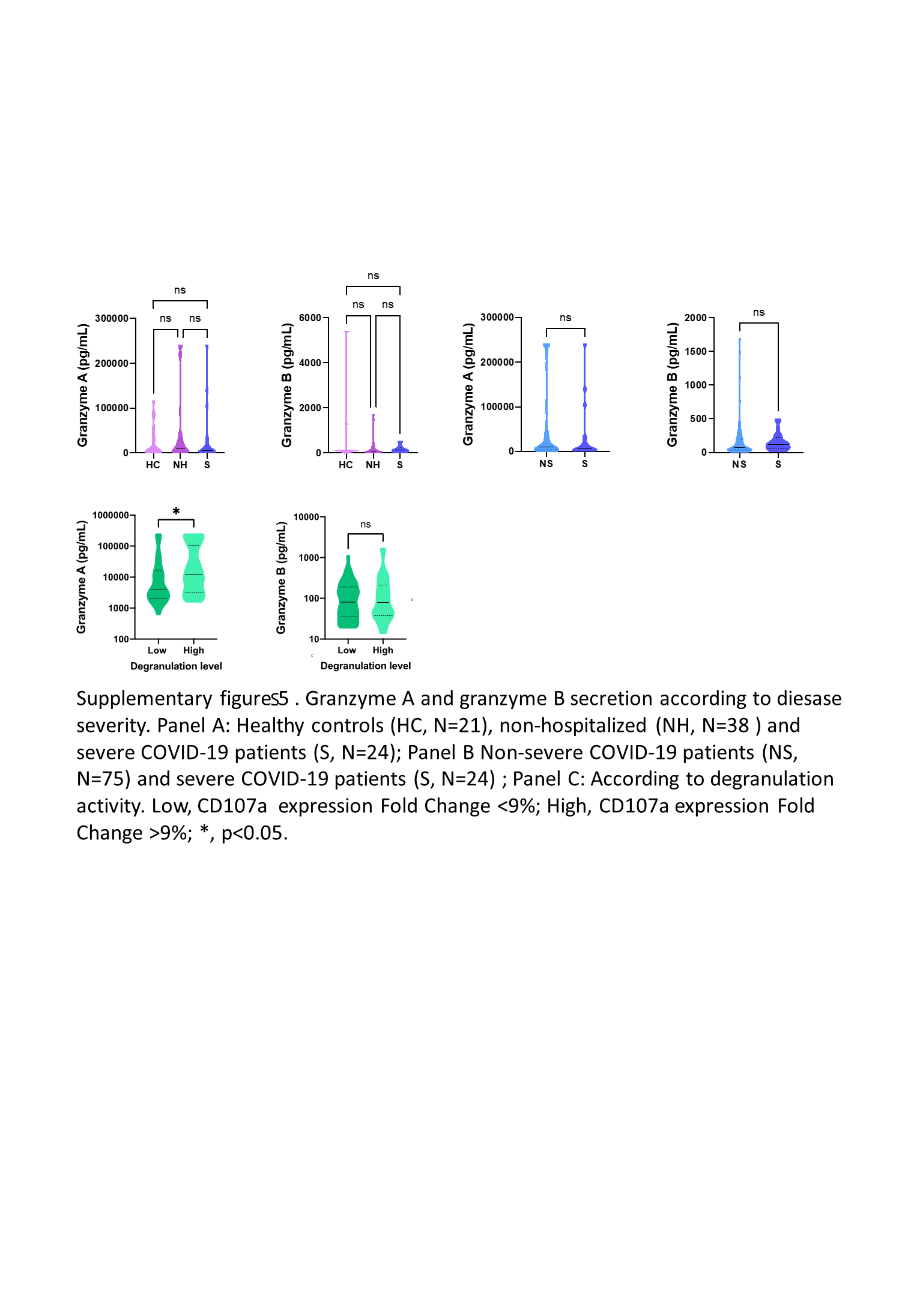

Supplement: Supplementary file 1 [file ijms-23-06577-s001.zip › Diapositiva5.TIF]

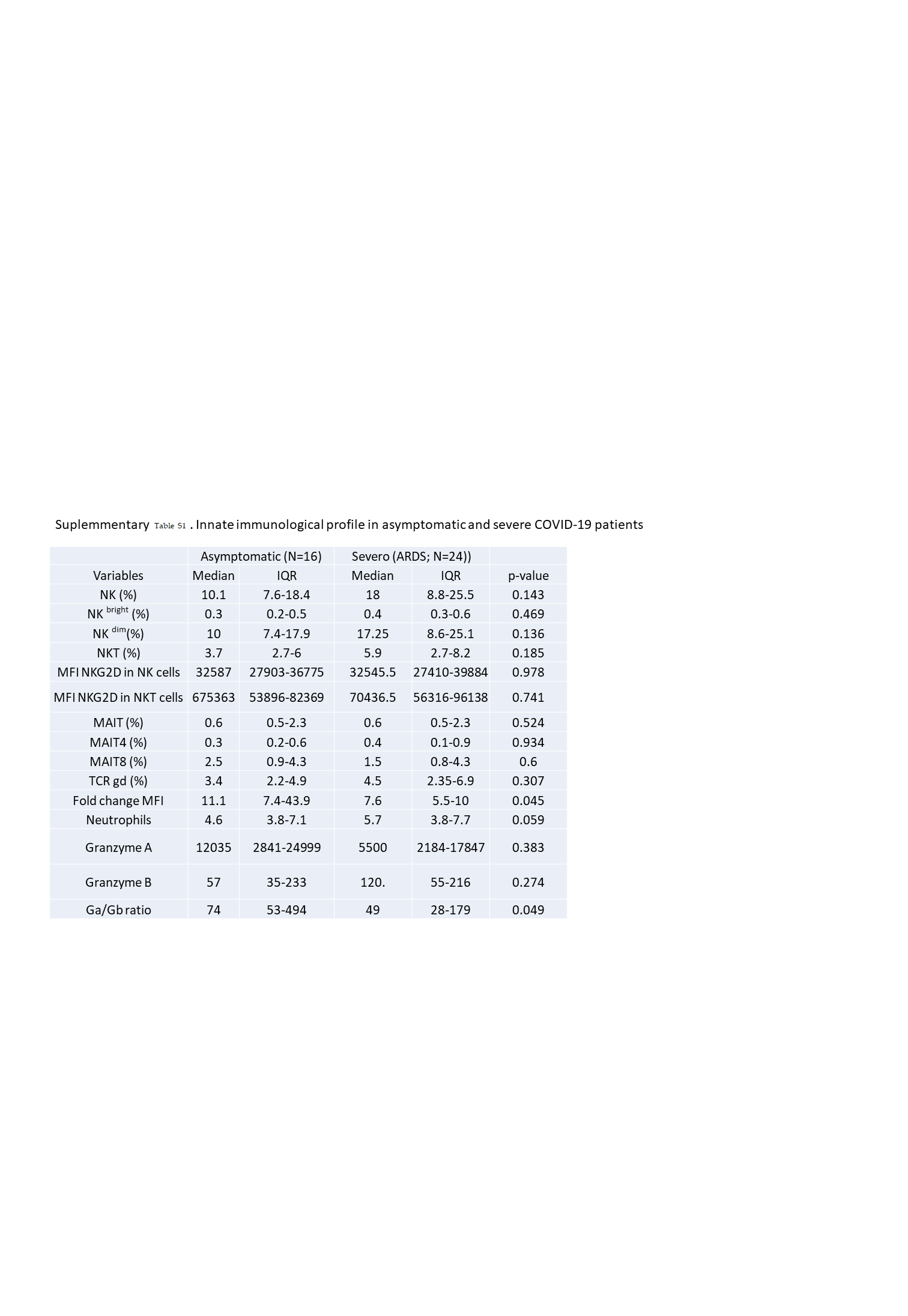

Supplement: Supplementary file 1 [file ijms-23-06577-s001.zip › Diapositiva6.TIF]

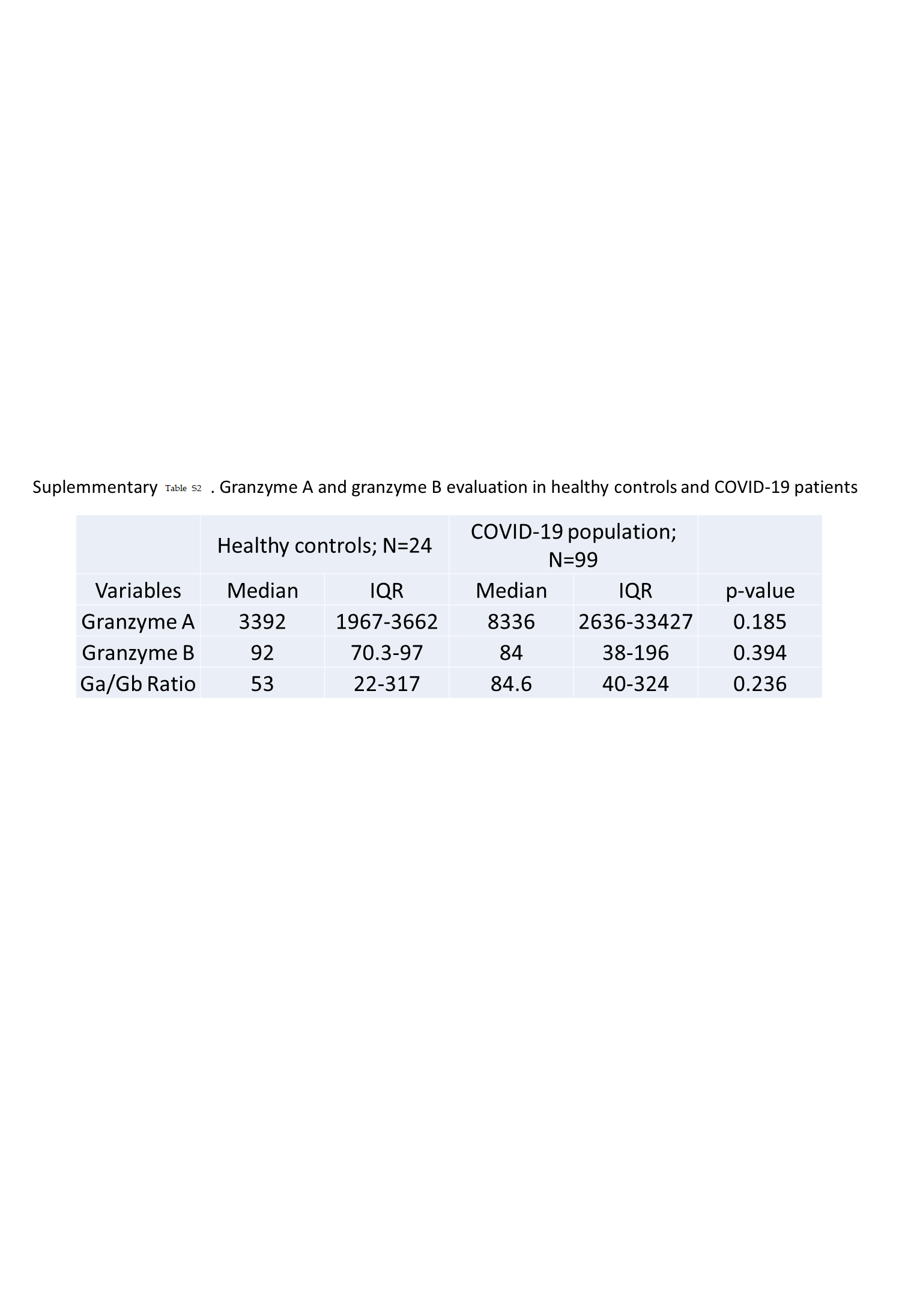

Supplement: Supplementary file 1 [file ijms-23-06577-s001.zip › Diapositiva7.TIF]
